# Cell Size Influences the Reproductive Potential and Total Lifespan of the *Saccharomyces cerevisiae* Yeast as Revealed by the Analysis of Polyploid Strains

**DOI:** 10.1155/2018/1898421

**Published:** 2018-03-20

**Authors:** Renata Zadrag-Tecza, Magdalena Kwolek-Mirek, Małgorzata Alabrudzińska, Adrianna Skoneczna

**Affiliations:** ^1^Department of Biochemistry and Cell Biology, Faculty of Biology and Agriculture, University of Rzeszow, Rzeszow, Poland; ^2^Institute of Biochemistry and Biophysics, Polish Academy of Sciences, Laboratory of Mutagenesis and DNA Repair, Warsaw, Poland

## Abstract

The total lifespan of the yeast *Saccharomyces cerevisiae* may be divided into two phases: the reproductive phase, during which the cell undergoes mitosis cycles to produce successive buds, and the postreproductive phase, which extends from the last division to cell death. These phases may be regulated by a common mechanism or by distinct ones. In this paper, we proposed a more comprehensive approach to reveal the mechanisms that regulate both reproductive potential and total lifespan in cell size context. Our study was based on yeast cells, whose size was determined by increased genome copy number, ranging from haploid to tetraploid. Such experiments enabled us to test the hypertrophy hypothesis, which postulates that excessive size achieved by the cell—the hypertrophy state—is the reason preventing the cell from further proliferation. This hypothesis defines the reproductive potential value as the difference between the maximal size that a cell can reach and the threshold value, which allows a cell to undergo its first cell cycle and the rate of the cell size to increase per generation. Here, we showed that cell size has an important impact on not only the reproductive potential but also the total lifespan of this cell. Moreover, the maximal cell size value, which limits its reproduction capacity, can be regulated by different factors and differs depending on the strain ploidy. The achievement of excessive size by the cell (hypertrophic state) may lead to two distinct phenomena: the cessation of reproduction without “mother” cell death and the cessation of reproduction with cell death by bursting, which has not been shown before.

## 1. Introduction

The *Saccharomyces cerevisiae* yeast has been one of the most frequently used model organisms in scientific studies, including studies of the mechanism of aging, as it was assumed that this mechanism is universal, at least for *Fungi* and *Metazoa* [1]. The contribution of yeast to such studies is based mainly on the analysis of replicative lifespan (RLS). This parameter is expressed as the number of daughter cells produced by a single “mother” cell during its life. This number is limited, as discovered by Mortimer and Johnston in 1959 [2], equaling an average of 20–30 generations. Having assumed that the number of daughters (buds) formed is a measure of the yeast cell's age, it was acknowledged that factors influencing that number are associated with regulation of the aging process, which is responsible for the limited replicative lifespan of the yeast cells. Thus far, the explanation of the phenomenon of limited reproductive potential of yeast cells has mainly been based on the “senescence factor” accumulation hypothesis [3]. Such an accumulation would lead to a progressive loss of reproductive capabilities by the “mother” cell. The molecules proposed as the “senescence factor” were primarily extrachromosomal rDNA circles [4], oxidatively damaged proteins [5], protein aggregates [6], or damaged mitochondria [7, 8]. Explanations of the phenomenon were also based on genetic regulation [9]. The aforementioned reasons for the limited reproductive capacity of yeast cells inevitably suggested that this phenomenon should be attributed to the aging process.

An alternative explanation that includes reasons unrelated to aging but that are still determined by cell genotype is offered by the hypertrophy hypothesis, which emphasizes cell size and its important role in the regulation of the reproductive potential of yeast [10, 11]. There exists a clear relationship between cell size and the number of daughter cells produced by a single yeast cell [12–15]. It is connected with an inevitable increase in the cell size observed in successive reproductive cycles, which is a consequence of the evolutionary selection of budding as the mechanism of cytokinesis. Such selection eliminates the possibility for reduction of the size of the mother cell, occurring in most eukaryotic cells in which cytokinesis results in two cells of equal sizes, where each of the cells is approximately half of the cell about to divide. Lack of cell size reduction results in the cell reaching the hypertrophy state after several dozen mitotic cycles, which can limit further reproduction [10]. The hypertrophy hypothesis postulates that the excessive size achieved by the cell—the hypertrophy state—is the reason that prevents the cell from further proliferation. Therefore, the value of reproductive potential is determined as the difference between the maximum size reached by the cell and the threshold value, which allows the cell to enter its first cell cycle, and the rate of the cell size increase per generation [10, 11]. It is worth pointing out that the aforementioned excessive cell size means that the maximum size prevents further reproduction. This maximum size is not universal and may vary depending on the genetic background, genetic changes, or environmental conditions. Therefore, the reproductive potential could be regulated by three parameters: the threshold cell size, the rate of the cell size increase per generation, and the maximum cell size [10, 11]. The relationship between the rate of the cell size increase per generation and the reproductive potential demonstrates that higher rates of cell size increase per generation lead to a decrease in the reproductive potential value and vice versa [15]. This relationship was also confirmed by other studies [12, 13, 16]. The hypothesis that the replicative lifespan of the *S*. *cerevisiae* yeast cells is not a direct consequence of the aging process is still a matter of debate [17, 18]. An important support for this approach may have come from recently published data showing the evidence of aging-independent causes of limited replicative lifespan in the symmetrically dividing *Schizosaccharomyces pombe* yeast [19]. However, further studies are needed to verify this hypothesis. Here, we present the results of a more comprehensive experimental approach, employing polyploid yeast cells.

In the natural environment, vegetative populations of the *S*. *cerevisiae* yeast appear in diploid or polyploid forms (autopolyploid and allopolyploid) [20]. In yeast, genome doubling is associated with morphological alterations of cell size and shape, colony organization and growth, generation time, and ecological tolerance. Metabolic changes are also observed, which are mainly associated with an increased rate of cell productivity [21]. The model based on polyploid yeast cells may also be helpful in explaining the causes of limited reproductive capacity of these cells because it provides another opportunity for the analysis of the phenomenon known as replicative aging. First, the use of isogenic cells—from haploid to tetraploid—makes it possible to compare these cells, as they contain the same genetic information but differ in the number of genome copies. Second, while these cells differ in ploidy, they also differ in average size [22]. Under optimum conditions, cell size may be doubled with the doubling of ploidy [23]. Although an increase in cell size may also be observed in the case of certain mutations or as a result of chemical factors, in these cases, such an increase is just one of many other consequences affecting the parameters in question. Another important reason is that in yeast, genome doubling is associated with metabolic changes, which can lead to an increased rate of cell productivity [21]. Because polyploids demonstrate increased productivity, the *Saccharomyces* polyploid has been studied from a biotechnological viewpoint, as many industrial yeast species used in bakery or brewery are polyploids [24, 25]. Therefore, the adopted model allows for a more precise assessment of the role of such factors as cell size or cellular biosynthetic potential in the regulation of the reproductive potential and total lifespan.

The goal of this study was to attempt to verify the role of cell size, both as a physical and physiological parameter, in determining the numeric value of the reproductive potential and its impact on the total lifespan of yeast cells. For that purpose, parameters such as reproductive potential and total lifespan, changes of cell size during the reproductive phase of life, and physiological and genetic parameters of an isogenic set of strains varying in ploidy—from haploid to tetraploid cells—were analyzed. Analyses were performed using three different genetic backgrounds to verify the phenomenon itself and determine its universality.

## 2. Materials and Methods

### 2.1. Yeast Strains and Growth Conditions

Yeast strains used in this study are listed in Table 1. Yeast cells were grown on standard liquid YPD medium (1% yeast extract, 1% Bacto Peptone, 2% glucose), unless stated otherwise. Cells were cultivated in a rotary shaker at 150 rpm or on solid YPD medium containing 2% agar, in the temperature of 28°C. In experiments, in which the selective conditions were necessary, the synthetic complete (SC) medium (0.67% yeast nitrogen base, 2% glucose, supplemented with amino acids, uracil, and adenine) was used.

### 2.2. Yeast Strain Construction

The strains YAS281 (1n), YMR2, YAS288 (*α* c:L), and YMS14 (2n) were prepared as described previously [26]. Briefly, YAS281 (called for simplicity in this paper BY474X (1n)) was obtained by reconstitution of functional *URA3* locus in BY4741 strain; YMR2 and YAS288 (*α* c:L) were obtained by disruption of *CAN1* locus with *can1::LEU2* cassette in BY4741 and BY4742, respectively. The diploid YMS14 strain (called later BY474X (2n)) was prepared by mating YAS281 (1n) and YAS288 (*α* c:L) strains. YAS228 diploid strain resulted from crossing YMR2 and BY4742 haploids of opposite mating types. DNA fragment carrying *can1::HIS3* deletion cassette was obtained by PCR-amplifying *HIS3* gene from pRS313 plasmid [27] with primers Can1K.up: 5′-CTTCAGACTTCTTAACTCCTGTAAAAACAAAAAAAAAAAAAGGCATAGCATCGGTGATGACGGTGAAAAC-3′ and Can1K.lw: 5′- AGAATGCGAAATGGCGTGGAAATGTGATCAAAGGTAATAAAACGTCATATAAAAACTTGATTAGGGTGAT-3′. This fragment was introduced into the YAS228 strain to obtain YAS250 strain. Triploid and tetraploid strains were prepared as follows: YAS250 strain was subject of protoplast fusion with YAS281 (1n) resulting in triploid YAS300 strain (i.e., BY474X (3n)). To create tetraploid strain YAS318 (i.e., BY474X (4n)), the protoplast fusion between cells of YMS14 (2n) and YAS250 strains was performed.

### 2.3. Protoplast Preparation for Fusion

Yeast strains were cultivated in 5 mL YPD medium with agitation at 28°C to the exponential phase (about 1 × 10^7^ cells/mL). The 3 mL aliquots of cell suspensions were spun down in the microcentrifuge (3000 rpm, 30 s) and washed twice with sterile water. Then, the cell pellets were suspended in 1 mL of buffer I (1 M sorbitol, 0.1 M EDTA); Zymolyase® 100T was added to the concentration 40 *μ*g/mL, and cells were incubated at 30°C for an hour. Resulting protoplasts were washed 2-3 times with 0.5 mL of protoplast buffer (0.1 M Pi pH 7.5, 0.8 M sorbitol), with gentle centrifugation (3000 rpm, 30 s) in between. Finally, the pellet was gently resuspended in 100 *μ*L of protoplast buffer.

### 2.4. Protoplast Fusion

The protoplast fusion was performed according to [28] with some modifications. Briefly, 100 *μ*L suspensions of protoplasts of fusion partners were mixed together and spun down (3000 rpm, 30 s) and after careful removing of the supernatant, the pellet was suspended in fusion buffer (35% PEG 3350, 10 mM CaCl_2_, and 0.8 M sorbitol) followed by 20 minutes incubation at room temperature. Then, the protoplasts were very gently centrifuged (1000 rpm, 3 min), washed 3 times with protoplasts buffer, and finally resuspended in 1.5 mL of the same buffer. Different volumes (0.1–1 mL) of the fused protoplast suspensions were gently mixed with 10 mL of regeneration medium (0.67% YNB medium, 0.8 M sorbitol, and 0.6% agar) and poured onto the surface of thin layer of synthetic minimal regeneration medium (0.67% YNB medium, 2% glucose, 0.8 M sorbitol, and 2.5% agar). Plates were incubated at 28°C for a week. The resulting colonies were isolated, and the DNA content in the cells of polyploid strains was confirmed by flow cytometry.

### 2.5. FACS Analysis

The DNA content of *S*. *cerevisiae* cells was measured by flow cytometry as previously described [29]. Additionally, an analysis of the size of the cells present in the analyzed population of 10,000 cells were performed.

### 2.6. Determining Reproductive Potential

Reproductive potential of individual yeast cells was determined by a routine procedure [30] on cells placed on agar plates using a micromanipulator. The reproductive potential of each cell was defined as the number of buds formed by the cell. Results of two independent experiments, each on at least 40 cells, were taken at each experimental point.

### 2.7. Determining Reproductive Potential and Reproductive, Postreproductive and Total Lifespan

Yeast lifespan (reproductive and postreproductive) was determined as described previously [31] with modifications described in Zadrag et al., [32]. Yeast cultures were grown on YPD liquid medium overnight. One-microliter aliquots of culture were dropped on YPD plates with solid medium containing Phloxine B at the concentration of 10 *μ*g/mL, for monitoring the moment of cell death. For each experiment, forty single cells were micromanipulated to the appointed area. The first daughters were chosen as the starting cells, and their successive buddings were followed to determine the reproductive potential and reproductive lifespan expressed in time units. After completion of the buddings, yeast cells were inspected in one-hour intervals to determine the moment of cell death and length of their postreproductive lifespan. Total lifespan was calculated as the sum of hours which cell spent in the reproductive and postreproductive phases of life. During the manipulation, the plates were kept at 28°C for 16 h and at 4°C during the night (8 h). The data represent mean values from two separate experiments.

### 2.8. ATP Content Estimation

ATP content was assessed with BactTiter-Glo™ Microbial Cell Viability Assay according to the manufacturer protocol (Promega). Cells were suspended in a 100 mM phosphate buffer with pH 7.0, containing 0.1% glucose and 1 mM EDTA. A sample (100 *μ*L) of cell suspension with the density of 10^6^ cells/mL was used for determination purposes. Luminescence was recorded after 5 min using a TECAN Infinite 200 microplate reader. The luminescent signal was proportional to the amount of ATP present, which was directly proportional to the number of cells.

### 2.9. Estimation of Glucose Consumption Rate

Quantitative determination of glucose concentration was performed with copper and molybdenum phosphate reagents by the Somogyi-Nelson method [33]. Glucose reduces the Cu^2+^ ions to Cu^+^ ions. The amount of copper oxide (I) was determined using the arsenomolybdate reagent, which is reduced to molybdenum blue. The intensity of the resulting blue color as a product is proportional to the amount of Cu_2_O and therefore the amount of glucose in the analyzed sample.

Yeast cells from the exponential phase of growth were adjusted to the density of 1 × 10^7^ cells/mL, collected by centrifugation (2 min, 7000 rpm) and resuspended in fresh complete medium. After 4 hours of incubation, the cell suspension was centrifuged and supernatant was used for the glucose concentration assay. Samples of media were diluted 100-fold to the volume of 0.5 mL; afterwards, 0.5 mL of the Somogyi-Nelson reagent was added to the media and placed in a boiling water bath for 20 minutes. After cooling of the samples, 0.5 mL of arsenomolybdate reagent was added, and the samples were incubated for 5 min. Then, 3.5 mL of water was added and allowed to stand for another 10 min. Absorbance of the samples was measured at *λ* = 520 nm. Glucose content was calculated from a standard curve made for samples with known concentrations of glucose.

### 2.10. Estimation of Relative RNA Level

Relative RNA content was measured by the method described in [34]. Acridine orange (3,6-dimethylaminopyridine) may bind with nucleic acids forming two types of complexes. The type A complex is formed when molecules of the dye intercalate between bases in a double-stranded DNA and double-stranded RNA fragments. Such a complex shows the green fluorescence. The type B complex is formed when particles of the dye will form aggregates of a single-stranded RNA or denatured single-stranded DNA, which gives the red fluorescence.

Yeast cells from the exponential phase of growth were adjusted to the density of 1 × 10^8^ cells/mL and collected by centrifugation (2 min, 7000 rpm). Cells thus obtained were fixed with 70% cold ethanol for 30 min at room temperature. After fixation, cells were washed twice with cold, sterile PBS and then suspended in a fresh buffer with no alteration in density. 200 *μ*L of cell suspension with a 400 *μ*L of permeabilisation buffer (0.1% Triton X-100, 80 mM HCl, and 150 mM NaCl) was mixed and incubated on ice for 2 min. Afterwards, 1.2 *μ*L of the acridine orange solution (concentration of 6 *μ*g/mL) was added and incubated at low temperature for 10 min. Cells were harvested by centrifugation and suspended in a small volume of PBS. Microscopic observations were carried out using the OLYMPUS BX-51 epifluorescence microscope equipped with the DP-72 digital camera and the cellSens Dimension software at *λ*_ex_ = 488 nm and *λ*_em_ = 650 nm. The images for all tested cells were performed at exactly the same parameters (the power of lamp and the exposure time) for possibility the comparison of fluorescence signal intensity. To determine the relative value of RNA, the multichannel images were separated into individual color channels (red, green, and blue). Fluorescence intensity (the sum of signal from pixels contained in the cell area) was measured only for the red color channel which corresponded to the relative value of RNA. Analysis was performed for at least 100 cells for each strain and for each biological replication. The quantitative results are presented as mean ± SD from at least three independent experiments.

### 2.11. Spontaneous Mutagenesis Assay

To determine the mutation frequency at *CAN1* marker gene, the forward mutation assay was employed, as described previously [35]. Briefly, yeast strains were cultured with agitation at 28°C to logarithmic growth phase (1-2 × 10^7^ cells/mL) in SC medium, then the number of Can^R^ clones were estimated by plating 100 *μ*L of the undiluted cultures on SC medium lacking arginine but containing 30 *μ*g/mL l-canavanine sulphate (Sigma). Mutant colonies were counted following incubation of the plates for 3 days at 28°C. To calculate the frequency of spontaneous mutations, the number of mutant colonies was normalized to the number of colonies grown on the control SC medium plates. In each experiment, 8 to 10 independent cultures of each yeast strain tested were analyzed. The presented data are the medians calculated from at least three separate experiments. Similar approach was applied to determinate the frequency of 5-FOA^R^ mutants, except that the *URA3* gene served in this assay as mutagenesis marker, and the selection of mutants was performed on SC media containing 1 g/L of 5-fluoroorotic acid (TRC, Canada).

### 2.12. Estimation of Cell Volume

Cell volume changes during the reproductive lifespan were estimated through an analysis of microscopic images recorded every fifth cell cycles during a routine procedure of reproductive potential determination. Images were captured with a Nikon Eclipse E200 microscope with 20x lens equipped with a digital camera. Cell diameter was measured four times in various planes for each cell using the MicroImage 3.0 software. For each yeast strain, at least 80 cells were counted. The mean cell volume in the population was estimated by analysis of the microscopic images recorded during the exponential phase of the cell growth. Images were captured with an Olympus BX51 microscope. Cell diameter was measured four times in various planes for each cell using the cellSens Dimension software. In all cases, the mean value of the cell diameter was used for the calculation of the cell volume. Cell volume was estimated using the following formula: *V* = 4/3π(*a*^2^*b*), where *a* and *b* are the radius calculated as a half of minor and major cell diameters, respectively.

### 2.13. Statistical Analysis

Statistical analysis of data was performed using the StatSoft Inc. (2011) STATISTICA data analysis software system, version 10.0 (https://www.statsoft.com). The statistical significance of differences between haploid strains (1n) and the 2n-4n strains was estimated using one-way ANOVA and Dunnett's post hoc test for SP4 and BY474X strains. The values were considered significant if *P* < 0.05. In the case of BMA64 yeast strains, the differences between the haploid and diploid strains were assessed using the *t*-test for independent samples.

## 3. Results

For the analysis, isogenic groups of yeast strains were prepared representing three independent genetic backgrounds: SP4, BY474X, and BMA64. With an exception of BMA64 yeast strains, each of the groups contained cells varying in the number of genome copies: haploid cells (1n), diploid cells (2n), triploid cells (3n), and tetraploid cells (4n). In the case of BMA64 yeast strains, the cells represented only haploid and diploid forms due to the very high instability of forms with ploidy levels higher than diploid and the quick reversion to the haploid state of such forms. To verify cell ploidy, DNA content was determined for each of the analyzed yeast strains (Figure S1). The purpose of analyses carried out with the use of three genetic backgrounds was to verify if the phenomenon is universal or whether it is closely dependent on the genetic background.

### 3.1. Reproductive Potential and Total Lifespan of Yeast Cells Differing in Ploidy

**Figure 1:**
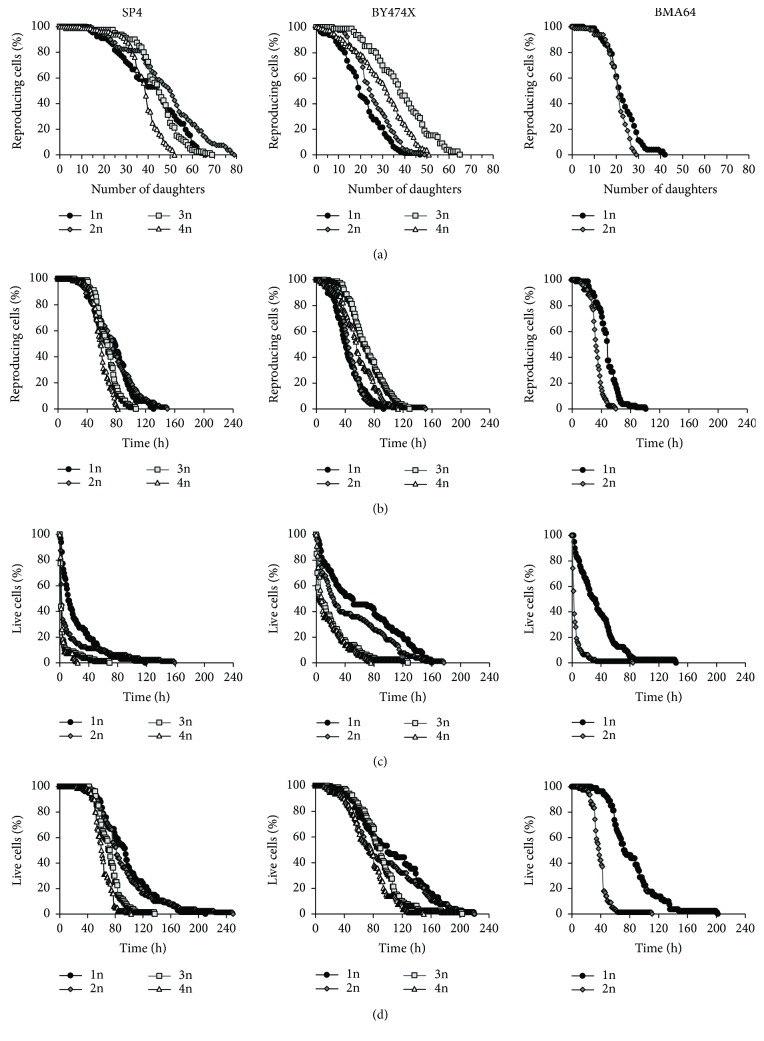
Reproductive potential and time of lifespan of yeast cells differing in ploidy. Budding lifespan (a), reproductive lifespan (b), postreproductive lifespan (c), and total lifespan (d) of yeast strains of different ploidy (from 1n to 4n) representing three genetic backgrounds: SP4, BY474X, and BMA64. Yeast cells during the experiments were grown on solid YPD medium containing 10 *μ*g/mL Phloxine B for cell viability monitoring. The data represent the mean values from two independent experiments of 40 cells each.

The analysis of the reproductive potential of cells differing in ploidy has shown that its value does not directly depend on the number of genome copies. This means that the reproductive capacity of the cell does not increase linearly along with the growth of genome copies. Moreover, the parameter values showed a distinct dependence on the genetic background, and the phenotype of each of the analyzed genetic backgrounds was slightly different. The reproductive potential of SP4 diploid and triploid cells was increased in comparison to haploid cells, but the value for tetraploid cells was clearly lower. Cells in the BY474X genetic background, ranging from diploid to tetraploid, have shown higher values of reproductive potential in comparison to haploid cells; however, the relation was not linear, as the value of the parameter for diploid and tetraploid cells was nearly the same. In contrast, the reproductive potential of diploid cells in the BMA64 genetic background was lower in comparison to haploid cells (Figure 1(a), Table 2).

The total lifespan consists of two phases, reproductive and postreproductive, and each of these phases may be regulated differently. When the reproductive phase of cell life was expressed in units of time (Figure 1(b); Table 2), differences in the reproductive potential between cells differing in ploidy were significantly lower than when the reproductive phase was expressed in the number of daughters produced by these cells (Figure 1(a); Table 2). The reproductive lifespan of diploid cells was comparable to that of haploid cells in both SP4 and BY474X genetic backgrounds. While for tri- and tetraploid SP4 cells, the value of the parameter was lower (the difference was statistically significant for tetraploid cells only); for tri- and tetraploid cells in the BY474X background, the value was significantly higher in comparison to haploid and diploid cells. The reproductive lifespan of diploid cells in the BMA64 genetic background was significantly shorter in comparison to haploid cells. The analysis of postreproductive lifespan (length of life after the end of reproduction phase) showed a negative correlation between the number of genome copies and the length of the postreproductive phase of a cell's life. In particular, in the case of tri- and tetraploid cells of SP4 and BY474X genetic backgrounds, the postreproductive lifespan was significantly shorter (ca. three or four times) in comparison to haploid cells. An extremely short postreproductive lifespan was observed in the case of BMA64 diploid cells: it was approximately seven times shorter in comparison to haploid cells. The shortening of the postreproductive lifespan in the case of diploid and polyploid cells (3n-4n) was observed for each of the analyzed genetic backgrounds (Figure 1(c); Table 2). The total lifespan of diploid cells of the SP4 and BY474X genetic backgrounds was comparable to that of haploid cells. A significant shortening of the postreproductive lifespan in the case of the tri- and tetraploid cells resulted in those cells having clearly shorter total lifespans in comparison to haploid or diploid cells. Similar results were obtained for the SP4 and BY474X genetic backgrounds. The biggest difference between the analyzed cells was observed in the case of the BMA64 genetic background, where the total lifespan of diploid cells was twice as short as that of haploid cells (Figure 1(d); Table 2). Considering the mean values and the shapes of the curves of the reproductive, postreproductive, and total lifespans, the analyzed cells fall into two groups: the first comprising haploid and diploid cells and the other tri- and tetraploid cells.

### 3.2. Physiological Parameters of Yeast Cells Differing in Ploidy

An increase in the number of genome copies may have an impact on increasing the cellular biosynthetic potential. Therefore, an analysis was performed of selected parameters that may have an influence on that potential. The ATP level of diploid cells was comparable to that of haploid cells, whereas for tri- and tetraploid cells, the values of the parameter were significantly higher (approximately eight times) in comparison to both haploid and diploid cells. Such a pattern was observed for both SP4 and BY474X genetic backgrounds. For the BMA64 yeast strains, statistically significant differences were already observed in the case of diploid cells (Figure S2A). The ATP level may be associated with the rate of glucose uptake, which is the main source of carbon for yeast cells. This parameter may also be used for estimation of cellular physiology and biosynthetic possibilities. Generally, cells with higher numbers of genome copies showed a higher rate of glucose uptake (as shown by the lower glucose concentration in the medium), but the statistical significance of the observed differences depended on the genetic background (Figure S2B). The haploid and diploid cells of the SP4 genetic background showed a similar rate of glucose uptake. In comparison to those cells, the rate of glucose uptake for tri- and tetraploid cells was significantly higher. In the case of the BY474X genetic background, a higher rate of glucose uptake was shown not only by tri- and tetraploid cells but also by diploid cells. Moreover, these differences compared to haploid cells were statistically significant. The differences in the rate of glucose uptake between haploid and diploid cells of the BMA64 genetic background were not statistically significant. Important information on the fitness of cells and their relative biosynthetic possibilities is also provided through the analysis of the relative RNA content (Figure S3). In each case, the fluorescence intensity indicating the relative level of RNA within the cell increased almost linearly with the increased ploidy. However, the differences were statistically significant in relation to haploid cells only for tri- and tetraploid cells from both SP4 and BY474X genetic backgrounds. For the BMA64 genetic background, the difference was already significant between the haploid and diploid cells (Figure S3D). As the observed relationship between cell ploidy and the relative RNA content might result from differences in cell size, the fluorescence intensity values were segregated by cell size. This approach, in which differences in cell size were considered, showed quite a different outcome. The relative RNA content in cells differing in ploidy was similar for all of the analyzed genetic backgrounds (Figure S3E).

### 3.3. The Spontaneous Mutagenesis Level in Yeast Cells Differing in Ploidy

**Figure 2:**
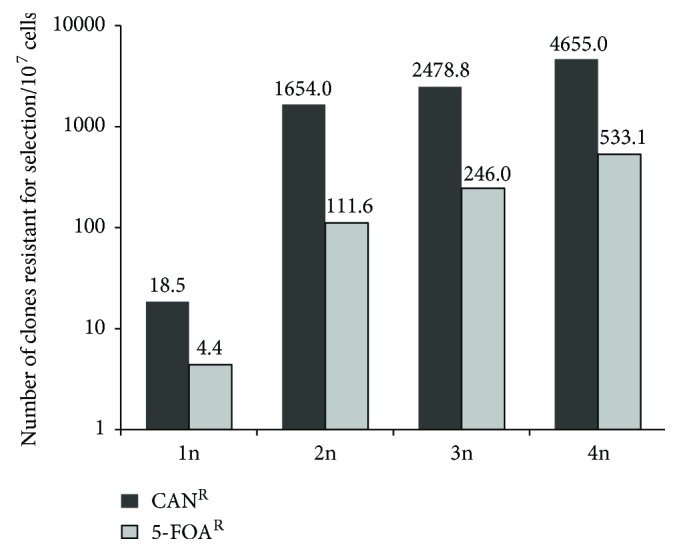
The spontaneous mutagenesis level in yeast cells depends on their ploidy. The changes in spontaneous mutagenesis levels at *CAN1* and *URA3* loci in cells of different ploidy (1n, 2n, 3n, and 4n) in the BY474X background. Spontaneous mutagenesis was measured using a forward mutation assay. Forward mutations leading to canavanine (Can^R^) or 5-fluoroorotic acid (5-FOA^R^) resistance are median values from at least 30 independent cultures of each strain in three separate experiments. The differences of mutation frequencies between investigated strains were statistically significant (*P* < 0.05).

Considering the significant shortening of the postreproductive and total lifespans, especially in the case of the tri- and tetraploid cells, we asked if discrepancies in the mutagenesis level could be responsible for such effects. For this reason, the spontaneous mutagenesis level was measured in all four strains from 1n to 4n in the BY474X genetic background. This experiment was only possible for these particular strains out of all strains analyzed in this work. To perform the forward mutation assay, the strain should possess the marker for mutagenesis in its genome. During the construction of BY474X strains, two such markers were introduced: *CAN1* and *URA3*. The presence in the given strain of only one functional copy of the gene, which serves as the mutagenesis marker locus (e.g., *CAN1* in haploid, *CAN1*/can1 in diploid, and *CAN1*/can1/can1 in triploid), allows tracking of the frequency of marker loss. Because *CAN1* encodes arginine permease, growing the cells on the SC medium devoid of arginine and containing canavanine, the toxic analog of arginine, permits positive selection of mutants. Similar tests could be performed using the *URA3* gene as a marker locus, except that the selection of mutants is performed on the SC medium supplemented with 5-fluoroorotic acid (5-FOA). The *URA3* gene encodes an orotidine-5′-phosphate decarboxylase, the enzyme that converts 5-FOA to toxic 5-fluorouracil. The results of the performed mutagenesis tests are presented in Figure 2. In addition to the already known difference of almost two orders of magnitude in mutation frequency between haploid and diploid strains [26], an additional increase in mutation frequency with the rising ploidy was detected. However, this increase was not as spectacular as between haploid and diploid strains; the mutation levels doubled with each additional genome copy.

### 3.4. Changes of Cell Size and Shape during the Reproductive Phase of Cell Lifespan

**Figure 3:**
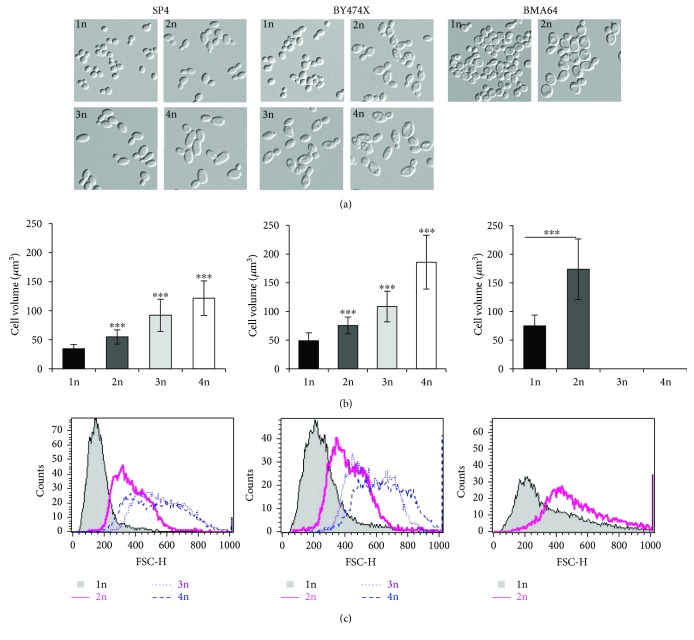
Mean cell size in a population of yeast cells differing in ploidy. (a) Shape and size diversity of yeast cells differing in ploidy. (b) Volume of yeast cells differing in ploidy. Cell volume was estimated by analysis of microscopic images. Data are presented as the mean values for at least 100 cells from each yeast strain. The bars indicate SD from all cells tested in two independent experiments. The stars indicate values that are significantly different from values obtained for the haploid strain (1n) within the same genetic background using one-way ANOVA and Dunnett's post hoc test for SP4 and BY474X strains or *t*-test for BMA64 strain; ^∗∗∗^*P* < 0.001. (c) Cell size of the yeast strains in the SP4, BY474X, and BMA64 backgrounds as measured by forward scatter (FSC histogram reflects the cells size in the population). The cells were analyzed via FACS as described in the Materials and Methods. Histograms were obtained for 10,000 cells per strain.

**Figure 4:**
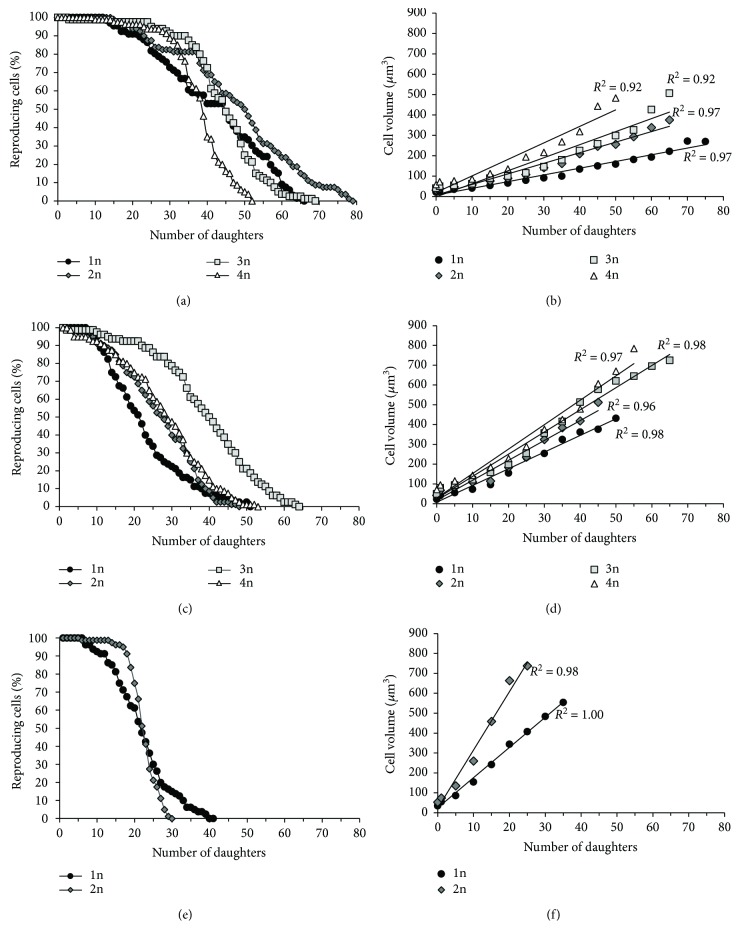
Changes in the size of individual yeast cells during the reproductive phase of life. Reproductive potential of the yeast strains differing in ploidy (from 1n to 4n) representing three genetic backgrounds (a) SP4, (c) BY474X, and (e) BMA64 was determined by the micromanipulation method. Changes in the cell size during the reproductive phase of yeast cell life were estimated by analysis of microscopic images recorded every fifth cell cycle during the determination of reproductive potential of (b) SP4, (d) BY474X, and (f) BMA64. Data were obtained from two independent experiments of 40 cells each.

**Figure 5:**
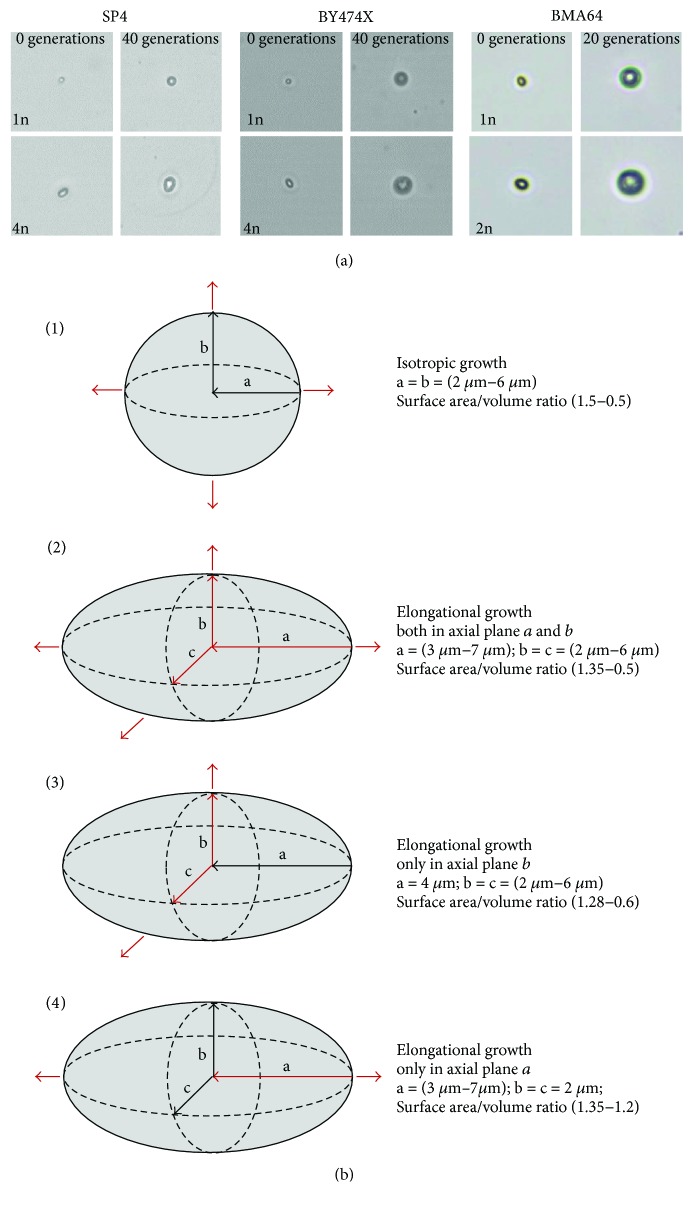
Morphological changes of the yeast cells during the reproductive phase of growth. (a) Changes in the shape of the yeast cell during the reproductive phase of life were assessed by analysis of microscopic images recorded during the reproductive potential determination procedure. The images are representative of all cells analyzed in two independent experiments. (b) The ratio of surface area and cell volume (A/V) changes depending on the shape and type of the cell growth. (1) The isotropic growth of spherical cells leads to fast decrease in the A/V ratio value. (2) The elongational type of growth of ellipsoidal cells when their size is increased in the two axial planes leads to fast decrease in the A/V ratio value. (3) The elongational type of growth of ellipsoidal cells when their size is increased only in axial plane b leads to fast decrease in the A/V ratio value. (4) For the elongational type of growth of ellipsoidal cells when growth occurs in one axial plane, the cell may maintain an almost steady value of the A/V ratio. The red arrows show a growth direction.

The analyzed yeast strains exhibited differences in cell morphology, which mainly concern cell size. In each of the analyzed genetic backgrounds, the mean cell size in a population increased in a straight line along with cell ploidy. The differences between haploids and cells of higher ploidy (from 2n to 4n) were statistically significant. Moreover, differences were noted regarding the values of the size parameter and the analyzed genetic backgrounds in the ascending sequence from SP4 through BY474X to BMA64 (Figure 3). However, the rate of cell size growth per generation was a more important factor for regulating the reproductive potential of yeast cells than the mean cell size in a population. The analysis of this parameter has shown a clear linear dependence (*R*^2^ in the 0.92 to 1 range) in the case of each strain. For each genetic background, the formula defining the behavior of individual cell types was the same, but the slope of the curves was different, revealing the differences in the growth rate per generation. The cells with higher ploidy showed a higher growth rate per generation. During production of subsequent buds, that is, during the reproductive phase, the cells significantly increased in size. The maximum values reached by the cells were dependent on the time of generation and cell size growth during a single cycle (Figures 4(b), 4(d), and 4(f)). The increase in size may also be accompanied by a change in the cell's shape. For haploid cells, a gradual increase in size was observed, although these cells maintained the same regular shape during the whole reproductive phase of life. By contrast, for diploid cells and especially for tri- and tetraploid cells, an increase in size was accompanied by a change in the shape from ellipsoidal, which was dominant for the major part of the reproductive phase, to spherical, which appeared at the end of that phase. Such changes were observed for the strains in both SP4 and BY474X genetic backgrounds. In turn, the BMA64 cells showed a rapid increase in cell size while maintaining a regular spherical shape (Figure 5(a), Figure S5).

## 4. Discussion

The phenomenon of replicative lifespan of the *Saccharomyces cerevisiae* yeast cells has been extensively analyzed but usually only in the context of the aging process. Furthermore, the replicative lifespan represents only a part of the total lifespan, which may be divided into two phases: the reproductive and the postreproductive [32]. Even though the most commonly used explanation for the limited reproductive capacity of these cells is the aging process, it can also be modulated by many aging-independent factors. In addition, both phases may be regulated by these factors through a common mechanism or each phase may be regulated by distinct factors [36]. Increase in genome copies can have an impact on (i) enhanced biosynthetic capacity (physiological efficiency), (ii) genetic stability, and (iii) cell size. Hence, studies based on polyploid cells may provide important information concerning the mechanism regulating the reproductive potential as well as the total lifespan.

### 4.1. Physiological Efficiency and Genetic Stability in Regulation of Reproductive Potential and Total Lifespan of the Cells

Increase in genome copy number results in an increase in cell productivity [25], which in turn suggests a connection with the reproductive potential. It was confirmed that doubling of the number of genome copies (comparison between haploid and diploid cells) leads to an increase in the reproductive capacities of the cell [9]. However, the experimental data obtained for cells with higher ploidy presented in this paper do not indicate that the reproductive potential increases proportionally to the number of the genome copies. Moreover, in this case, the genetic background may also be an important factor (Figure 1(a)). Indeed, diploid and triploid cells do increase their reproductive potential relative to haploid cells; tetraploid cells, however, exhibit quite different behavior. The reproductive potential of these cells can be lower than that of haploid cells or, at best, reach the values achieved by diploid cells depending on the genetic background. Especially intriguing is the significant decrease of the reproductive potential of the BMA64 diploid cells in contrast to the increase of the values of this parameter for the SP4 and BY474X diploid cells (Figure 1(a); Table 2).

Increased numbers of genome copies increase general biosynthetic capabilities, which are an adaptation to the higher demand for cellular constituents resulting from the increase in cell size. Under optimum conditions, the mean cell size in the population doubles with the doubling of ploidy [22, 23], which was also confirmed by the results obtained in these studies (Figure 3). Analysis of biochemical parameters, such as uptake and metabolism of glucose and intracellular ATP levels, which refer to energy metabolism, and the total RNA level referring to the translation efficiency, indicates that increased values of these parameters correlate with increased ploidy, although not always in a proportional manner (Figures S2 and S3). On one hand, the increase of general biosynthetic capabilities, coupled with the increase in the number of genome copies, may be a result of the coordination of intracellular processes to ensure stability in terms of energy and metabolism and their adaptation to the needs arising from the increased cell size [37–40]. On the other hand, such increase may improve the reproductive capacity of cells, especially in the case of tri- and tetraploid cells, as almost 100% of the cells retain the ability to reproduce. Examples of this can be observed mainly at the beginning of the reproductive phase, when di-, tri-, and tetraploid cells have a significant dominance compared to haploid cells (Figures 1(a) and 4, Figure S4). However, this initial dominance quickly wanes and is followed by a clear and rapid decline in the reproductive possibilities of these cells. Such cell behavior may be because the biosynthetic-physiological efficiency of the cells has a significant effect on the reproductive potential but only up to the point at which another factor becomes critical for further reproduction. For explaining this phenomenon, two factors should be considered: genetic stability and cell size.

Increasing the number of genome copies may be beneficial, among other reasons, because of (i) cell protection in the case of damage of one of the gene copies (gene redundancy) [41]; (ii) higher growth rate; and (iii) greater adaptability, including but not limited to higher frequency of beneficial mutations and stronger fitness effects [42]. It is also associated with certain disadvantages that are mainly connected with cell division problems, increased chromosome instability, and DNA repair defects [23, 43], reviewed in [44]. Polyploidization may affect the genetic stability of the cell. Although the frequency of point mutations (such as frameshifts, transversions, or transitions) is the same for haploid and diploid cells, there is as high as a hundredfold difference in the frequency of spontaneous mutagenesis between these cells [26, 45]. This difference is due to various DNA rearrangement events that lead to loss of heterozygosity in diploid strains (namely, gene conversion, allelic crossover, and chromosome loss) [45, 46]. The analysis of spontaneous mutagenesis levels in triploid and tetraploid cells, measured by the frequency of *CAN1* or *URA3* marker loss performed in this work, revealed further increase in spontaneous mutagenesis concomitant with rising cell ploidy (Figure 2). Thus, increases in spontaneous mutagenesis, that is, higher genome instability, likely contribute to the viability of the cells. Surprisingly, a clear correlation between mutagenesis level and reproductive potential of the yeast cells and their survival rates cannot be seen. However, the mutagenesis frequency can be counted only per viable cells in an analyzed population. Therefore, it is highly probable that mutagenesis levels are in fact higher, but some mutagenic events remain invisible because their effects are lethal.

In comparison to haploid cells, tetraploid cells are no more sensitive to various environmental stresses [47]. This is likely because DNA lesions induced by environmental stress that affects an essential gene frequently lead to cell death in haploids, while in diploids or in polyploids, the redundant functional allele of the affected gene is available from another copy of the genome. On the molecular level, the accumulation of DNA lesions provoked by environmental stress stimulates DNA damage response, which encompasses not only recruitment of proper DNA repair proteins to the lesion but also activation of DNA damage checkpoint and cell cycle arrest [48, 49]. If the DNA damage is not repaired properly until mitosis (G2/M checkpoint), it can lead to permanent cell cycle arrest that concludes with cell death (via, e.g., mitotic catastrophe) [44]. That certainly influences, although adversely, both the reproductive potential of the cell and its postreplicative lifespan. The difference in the spontaneous mutagenesis level between haploids and diploids in BY474X background is vast (hundredfold), while between diploids and triploids or tetraploids, it is much lower (about twofold and fourfold, resp.); conversely, a remarkable difference in the reproductive potential is visible between diploids and haploids but not between diploids and tetraploids in this background. This inconsistency of results suggests that it is not genomic instability that limits the reproductive potential of the analyzed cells. However, it is also worth noting that even if no clear relationship is observed when particular factors are considered individually (e.g., impact of point mutation frequency on reproductive potential), their interactions and the resulting additive effect cannot be ruled out. This point of view is similar to that presented by Kaya et al. [50] with regard to mutation accumulation as a cause of haploid yeast aging. The authors emphasize the role of cumulative damage of nuclear and mitochondrial genomes, as opposed to individual damage types, in the regulation of the aging process. For that reason, it is highly probable that both genomic instability and cell size (hypertrophy) may contribute to the decrease of the cell's reproductive potential.

### 4.2. Cell Size as a Regulator of Reproductive Potential and Total Lifespan

Cell size is an important biological trait that both affects the internal architecture of the cell and determines the range of intracellular biological processes. Increase in cell size results in the need to adjust individual cell components (organelles and macromolecules) to keep them in the right proportions appropriate to the size of the cell [38, 51]. Exceeding a certain size limit may generate problems inter alia with intracellular transport and ensuring of appropriate level of signaling or regulating molecules (e.g., G1 phase cyclin level) necessary to continue reproduction. Processes such as cell growth and cell division are usually closely coordinated to keep the size of a given cell type constant. However, in the case of yeast, the cell size increases along with the subsequent reproductive cycles. This phenomenon is the consequence of budding and the lack of the ability of the cell to reduce its own size, favoring the achievement of an excessive size that makes further reproduction impossible.

The analysis of the reproductive potential of yeast cells shows that the mean value of this parameter does not increase linearly along with the increase in the number of genome copies (Figures 1(a) and 4). However, if we look at the shape of the survival curve, we may notice that along with an increase in ploidy, the part of the whole reproductive phase when almost 100% cells maintain ability to reproduce also increases (Figure 1(a), Figure S4). This suggests that in the absence of other regulators affecting reproductive capabilities of cells, the relationship between ploidy (which corresponds to the biosynthetic abilities of the cells) and the reproductive potential of the cell should be linear. Lack of such a relationship suggests a significant contribution of another factor, such as cell size, in the regulation of the reproductive potential. When analyzing the potential impact of cell size on the regulation of reproductive potential, it is worth noting the values of the threshold cell size, the rate of the cell size increase per generation, and the maximum cell size, since these values appear to be dependent on the biosynthetic capabilities of cells. This is indicated by the slope of the curve (Figure 4), which shows the rate of an increase in cell size per generation under the reproductive phase of life. These results also emphasize that there is no universal or exact value of cell size causing reproduction cessation because both the maximum cell size and the rate of achievement of the hypertrophy state may be modified through either genome changes or environmental conditions. The maximum cell size allowing for reproduction is higher for tri- and tetraploid cells in comparison to that for haploid and diploid cells (Figure 4), which corresponds to the biosynthetic abilities of these cells and explains the higher reproductive capacity of these cells (except for tetraploid cells). With regard to cells with a significant reduction of the reproductive potential, as in the case of tetraploid cells of the SP4 and BY474X genetic backgrounds and especially the diploid cells of the BMA64 genetic background, a rapid reduction in reproduction capabilities (these cells exhibited high fitness during almost 2/3 of their entire reproductive phase) may actually result from the cell achieving its critical size not only in terms of reproduction purposes but also in terms of cell integrity, which ultimately leads to frequent cell lysis (Figure 4, Figure S4). However, the increased levels of genomic instability due to ineffectiveness of DNA damage response or due to division problems (which might be the case for BMA64 genetic background, which shows signs of endoduplication) (Figure S1) may have enhanced this effect by influencing the rate at which the cells achieved the hypertrophy state.

Cell size is, on the one hand, a physical parameter that, by the surface to volume ratio, determines the size of the cell; on the other hand, it undoubtedly influences the internal architecture and physiological capacity of the cell. The studies conducted demonstrate the existence of two size thresholds: the first leads to cessation of reproduction, but cells are still alive; the second leads to cell death caused by bursting and cell lysis. Hence, cell size may affect not only the reproductive potential but also the total lifespan of cells by shortening the postreproductive phase (Figures 1 and 4). The two size thresholds are related to two distinct phenomena. One is of a physiological nature, because many cellular processes depend directly on cell size, for example, transport across membranes and inside the cell, biosynthetic reactions, and, importantly, the cell cycle. The cell cycle requires the cooperation of many mechanisms to maintain growth homeostasis and optimum cell size. It is therefore possible that a large cell size could affect this cooperation and therefore the ability of cells to reproduce. The other phenomenon occurs because of the obvious lack of strength of the “cell envelope,” that is, cell membrane (the lipid composition has an impact on the physicochemical properties) and cell wall. Mechanical strength of the cell wall relies on *β*-glucan and chitin [52]. During cell growth, the composition of *β*-glucan increases, which in turn causes an increase in cell wall thickness [53]; however, wall thickness does not affect the elastic properties [54]. Hence, the changes of the internal tension may have an effect on maintaining the integrity of the cell. Diploid BMA64 cells, which show the shortest postreproductive phase, can be used as an example: bursting of these cells occurs almost immediately after the last mitotic cycle. Cell bursting involves almost 90% of all analyzed diploid cells and many haploid cells of these strains. This phenomenon may also explain the behavior of tetraploid yeast cells, which also show a very short postreproductive phase. These cells reach the critical size for reproduction and subsequently the critical size for maintenance of cell integrity faster than haploid cells, which is why their total lifespan is shorter than that of haploid cells (Figures 1 and 4).

When analyzing the causes of cell achieving the size critical for maintaining cell integrity, attention should also be paid to morphological aspects of the cell, that is, cell shape and type of cell size increase. Cells that achieve the size that is critical for their integrity, especially BMA64 strain cells, show a relatively high increase in size during a single cell cycle (slope of the curves, Figure 4(f)). In addition, these cells maintain a very regular spherical shape, which does not change throughout their reproductive and postreproductive phases. In turn, the shape of polyploid yeast cells is more elliptical due to ploidy-dependent cytoskeletal organization. However, this shape may be changed to one that is more spherical, which occurs at the end of the reproductive phase of life (Figures 3(a) and 5(a), Figure S5). Maintenance of a proper surface area and cell volume ratio (A/V ratio) seems to protect the cell against loss of integrity (cell lysis). In general, the A/V ratio is inversely correlated with cell ploidy [55]; however, without cell elongation, the decrease in this ratio might be higher and might lead to reduction of cell functionality. This is confirmed by the data obtained from the analysis of the A/V ratio of cells of different shapes and types of cell size increase. The isotropic growth of spherical cells leads to a rapid decrease in the A/V ratio value. A similar pattern is noted for ellipsoidal cells, whose size is increased in two axial planes. Only when elongational growth occurs in one axial plane may the cell maintain an almost steady value of the A/V ratio (Figure 5(b)). The results obtained by Müller [22] do not indicate that the A/V ratio is the only factor responsible for limiting the reproductive potential of the cells but do not rule out its impact as one of the factors. In turn, the results presented in this paper point to a relevant impact of this parameter to maintenance of proper functionality and cell integrity and therefore the regulation of the reproductive potential and cell death.

**Figure 6:**
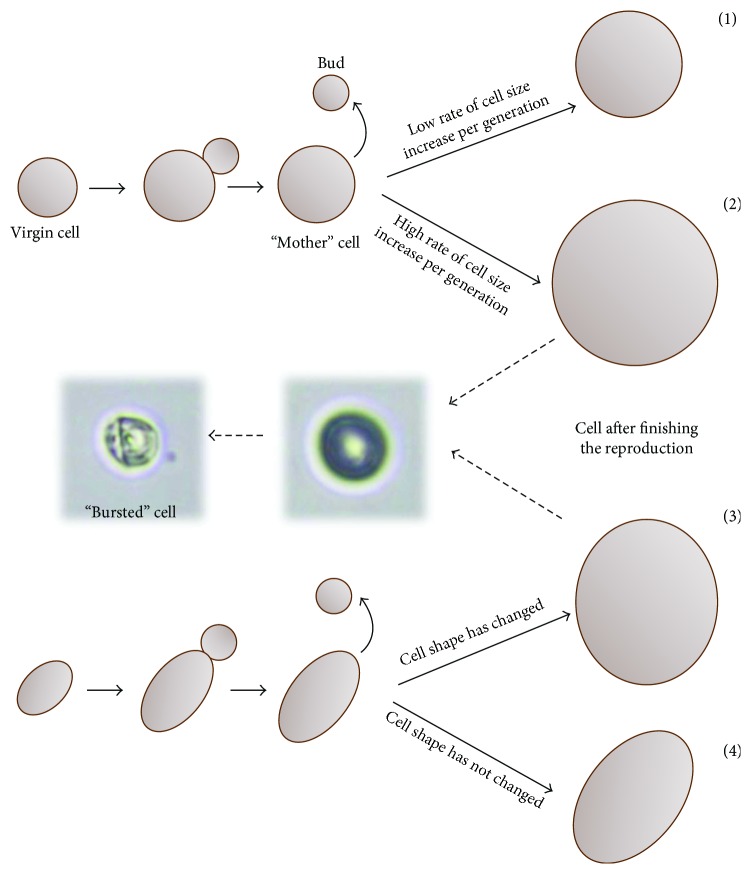
Possible size and shape changes of yeast cells during the reproductive phase of life. (a) The spherical cell with a regular shape. Cell size increases gradually, but its shape does not change during the reproductive phase of life. Depending on the rate of cell size increase per generation, spherical cells may reach the size that is critical only for further reproduction (no cell lysis occurs) (1) or that is also critical for the strength of the cell wall (cell lysis generally occurs) (2). (b) The ellipsoidal cell. Cell size increases gradually during each subsequent reproductive cycle. At the end of the reproductive phase, a cell may change its shape from elliptical to spherical (cell lysis may occur) (3) or maintain the elliptical shape (no cell lysis occurs) (4).

Based on these calculations and the observation of the morphological changes of cells during the reproductive phase (Figure 5, Figure S5), two different types of behavior depending on the cell shape may be proposed: (1) the spherical cell, which does not change its shape during the reproductive phase of life; depending on the rate of cell size increase per generation, spherical cells may reach the size that is critical only for further reproduction, for example, haploid cells, or also critical for maintaining cell integrity, for example, diploid cells from the BMA64 strain; (2) the ellipsoidal cell, which at the end of the reproductive phase may either change its shape from elliptical to spherical or maintain the elliptical shape (Figure 6). That latter alteration of the cell shape may favor a more rapid achievement of the size critical not only for reproduction but also for maintaining cell integrity. Therefore, in this case, cell lysis may occur, as in the case of tri- and tetraploid cells. The tensile strength of cell walls appears to be lower when the cell assumes a spherical shape. The ellipsoidal cell, even of the same volume as the spherical cell, is better protected against bursting, as it is still able to increase its smaller diameter. The phenomenon of cell bursting was also observed in the case of the *Sc*. *pombe* yeast cells. For those cells, the change of the cell shape from ellipsoidal to spherical was observed after several mitotic cycles, which also resulted in loss of integrity and cell lysis [56].

The analysis performed with the use of polyploid cells shows a relevant impact of cell size on the regulation of reproductive potential and total lifespan of yeast cells. The total lifespan analysis of cells with the higher-than-haploid number of genome copies has never been performed; therefore, it is an important aspect of discussion on the regulatory role of cell size. The postreproductive phase has a particular influence on the total lifespan [57]. As also shown in these studies, the length of that phase shows an inverted relationship with cell ploidy and is particularly short in the case of tri- and tetraploid cells (or even diploid cells of the BMA64 strain), which may be due to the cell size. This does not mean that accumulation of damage, genetic stability, or changes resulting from the passage of time does not play any role in the regulation of reproductive potential. However, the cessation of reproduction will largely be determined by whichever factor is first to achieve its critical value. Moreover, their additive effect cannot be ruled out, which may enhance the rate at which the cells achieve the hypertrophy state. The maximum cell size value, which leads to cessation of reproduction, can be regulated among others by biosynthetic possibilities; therefore, its value may differ depending on the number of genome copies. Moreover, the achievement of an excessive size (hypertrophic state) may lead to two distinct phenomena: cessation of reproduction without cell death and cessation of reproduction with cell death by cell bursting.

## Figures and Tables

**Table 1:** Yeast *S*. *cerevisiae* strains used in this study.

Strain	Genotype	Reference
SP4 (1n)	*MATα leu1 arg4*	(Bilinski et al, 1978)

SP4 (2n)	*MAT*a/*MATα leu1/leu1 arg4/arg4*	Lab collection

SP4 (3n)	*MAT*a/*MAT*a/*MATα leu1/leu1/leu1 arg4/arg4/arg4*	Lab collection

SP4 (4n)	*MAT*a/*MAT*a/*MATα*/*MATα leu1/leu1/leu1/leu1 arg4/arg4/arg4/arg4*	Lab collection

BY4741	*MAT*a *his3Δ1 leu2Δ0 met15Δ0 ura3Δ0*	*EUROSCARF*

BY4742	*MATα his3Δ1 leu2Δ0 lysΔ0 ura3Δ0*	*EUROSCARF*

BY4743	*MAT*a/*MATα his3Δ1/his3Δ1 leu2Δ0/leu2Δ0 LYS2/lys2Δ0 met15Δ0/MET1 ura3Δ0/ura3Δ0*	*EUROSCARF*

YAS288 (*α* c:L)	*MATα his3Δ1 leu2Δ0 lysΔ0 ura3Δ0 can1::LEU2*	(Alabrudzinska et al., 2011 [26])

YAS228	*MAT*a/*MATα his3Δ1/his3Δ1 leu2Δ0/leu2Δ0 LYS2/lys2Δ0 met15Δ0/MET15 ura3Δ0/ura3Δ0 can1::LEU2/CAN1*	This work

YAS250	*MAT*a/*MATα his3Δ1/his3Δ1 leu2Δ0/leu2Δ0 LYS2/lys2Δ0 met15Δ0/MET15 ura3Δ0/ura3Δ0 can1::LEU2/can1::HIS3*	This work

YMR2	*MAT*a *his3Δ1 leu2Δ0 met15Δ0 ura3Δ0 can1::LEU2*	(Alabrudzinska et al., 2011 [26])

YAS281 BY474X (1n)	*Mat*a *his3Δ1 leu2Δ0 met15Δ0 URA3*	(Alabrudzinska et al., 2011 [26])

YMS14 BY474X (2n)	*MAT*a/*MATα his3Δ1/his3Δ1 leu2Δ0/leu2Δ0 LYS2/lys2Δ0 met15Δ0/MET15 URA3/ura3Δ0 CAN1/can1::LEU2*	(Alabrudzinska et al., 2011 [26])

YAS300 BY474X (3n)	*Mat* a*/MAT*a/*MATα his3Δ1/his3Δ1/his3Δ1 leu2Δ0/leu2Δ0/leu2Δ0 LYS2/LYS2/lys2Δ0 met15Δ0/met15Δ0/MET15 ura3Δ0/ura3Δ0/URA3 can1::LEU2/can1::HIS3/CAN1*	This work

YAS318 BY474X (4n)	*Mat* a*/MAT*a/*MATα*/*MATα his3Δ1/his3Δ1/his3Δ1/his3Δ1 leu2Δ0/leu2Δ0/leu2Δ0//leu2Δ0 LYS2/LYS2/lys2Δ0/lys2Δ0 met15Δ0/met15Δ0/MET15/MET15 ura3Δ0/ura3Δ0/ura3Δ0/URA3 can1::LEU2/can1::LEU2/can1::HIS3/CAN1*	This work

BMA64-1A (1n)	*MAT*a *ura3-52 trp1Δ2 leu2-3112 his3-11 ade2-1 can1-100*	*EUROSCARF*

BMA64 (2n)	*MAT*a/*MATα ura3-52/ura3-52 trp1Δ2/trp1Δ2 leu2-3112/leu2-3112 his3-11/his3-11 ade2-1/ade2-1 can1-100/can1-100*	*EUROSCARF*

**Table 2:** The budding lifespan (number of generations), reproductive lifespan, postreproductive lifespan, and total lifespan of the yeast strains of different ploidy 1n–4n. Data are presented as the means ± SD from all tested cells during two independent experiments (80 cells). ^∗^*P* < 0.05; ^∗∗^*P* < 0.01; and ^∗∗∗^*P* < 0.001 compared to haploid (1n) strain (one-way ANOVA and Dunnett's post hoc test).

Yeast strain		Budding lifespan	Reproductive lifespan	Postreproductive lifespan	Total lifespan
		Number of daughters	Time (h)	Time (h)	Time (h)
SP4	1n	41.9 ± 14.9	73.2 ± 26.0	22.5 ± 25.5	95.7 ± 36.3
	2n	48.4 ± 16.3^∗∗^	75.6 ± 28.0	15.3 ± 31.4	90.7 ± 40.8
	3n	44.7 ± 10.6	66.3 ± 15.2	5.1 ± 11.3^∗∗∗^	71.4 ± 16.6^∗∗∗^
	4n	38.3 ± 7.8	58.3 ± 12.4^∗∗∗^	3.3 ± 5.0^∗∗∗^	61.6 ± 12.8^∗∗∗^

BY474X	1n	20.8 ± 9.7	43.1 ± 19.7	63.0 ± 51.5	106.1 ± 49.2
	2n	26.1 ± 8.7^∗∗^	47.5 ± 19.7	46.0 ± 46.8^∗^	93.5 ± 50.5
	3n	37.9 ± 14.1^∗∗∗^	73.6 ± 27.9^∗∗∗^	23.4 ± 29.9^∗∗∗^	96.9 ± 34.4^∗^
	4n	30.5 ± 12.3^∗∗∗^	56.3 ± 24.1^∗∗∗^	17.1 ± 20.3^∗∗∗^	73.43 ± 28.5^∗∗∗^

BMA64	1n	23.0 ± 7.0	47.4 ± 15.1	35.3 ± 29.3	82.7 ± 33.2
	2n	21.0 ± 5.6^∗^	32.9 ± 9.3^∗∗∗^	5.4 ± 10.8^∗∗∗^	38.3 ± 12.8^∗∗∗^

## Abbreviations

WGD: Whole-genome duplication
RLS: Replicative lifespan.

## Supplementary Materials

Supplementary Materials Figure S1: DNA content of yeast cells differing in ploidy. Cellular DNA content of the yeast strains in the SP4, BY474X, and BMA64 genetic backgrounds. Propidium iodide-stained cells were analyzed via FACS as described in the Materials and Methods. Histograms were obtained for 10,000 cells per strain. Figure S2: ATP content and glucose uptake in yeast cells differing in ploidy. (A) ATP content was determined using BactTiter-Glo Microbial Cell Viability Assay. Luminescence was recorded using the microplate reader. The luminescent signal corresponded to the ATP content, which was directly proportional to the number of cells. (B) Glucose concentration was determined in YPD medium supplemented with glucose (20 mg/mL) using the Somogyi-Nelson method. The YPD medium was collected after 4 hours of cell incubation and used for analysis. Absorbance was recorded using the microplate reader at *λ* = 520 nm. The results are presented as the mean values from three independent experiments. The bars indicate SD. The stars indicate values that are significantly different from values obtained for haploid strain (1n) within the same genetic background using one-way ANOVA and Dunnett's post hoc test for SP4 and BY474X strains or *t*-test for BMA64 strain; ^∗^*P* < 0.05; ^∗∗^*P* < 0.01; and ^∗∗∗^*P* < 0.001, respectively. Figure S3: comparison of relative RNA content in yeast differing in ploidy. Relative RNA content of yeast cells was assessed with acridine orange. Fluorescence was examined under the fluorescence microscope at *λ*_ex_ = 488 nm and *λ*_em_ = 650 nm. For determination of the relative value of RNA, images were separated into individual color channels; fluorescence intensity was measured only for the red channel using the cellSens Dimension software: (A) overlay; (B) red channel; (C) green channel. The results are presented as (D) the mean values of the fluorescence intensity for at least 100 cells from three independent experiments or as (E) calculations of the fluorescence intensity per cell size. The bars indicate SD. The stars indicate values that are significantly different from values obtained for haploid strain (1n) within the same genetic background using one-way ANOVA and Dunnett's post hoc test for SP4 and BY474X strains or *t*-test for BMA64 strain; ^∗^*P* < 0.05; ^∗∗^*P* < 0.01; and ^∗∗∗^*P* < 0.001, respectively. Figure S4: the impact of the number of genome copies on the reproductive possibility of the yeast cells. Calculations illustrating the part of the whole reproductive phase of life when almost all cells (90–100 percent) maintain the ability to reproduce. This value is strictly dependent on the number of the genome copies. Figure S5: changes in yeast cell shape during the reproductive phase of growth. Changes in the shape of the yeast cell during the reproductive phase of life were assessed by analysis of microscopic images recorded every fifth cell cycle during the reproductive potential determination procedure. The images are representative of all cells analyzed in two independent experiments. The analyzed yeast strains differ in ploidy (from 1n to 4n) and represent three genetic backgrounds: SP4, BY474X, and BMA64.
