# Supplementary material for: Cell Size Influences the Reproductive Potential and Total Lifespan of the Saccharomyces cerevisiae Yeast as Revealed by the Analysis of Polyploid Strains
Source: Oxid Med Cell Longev. 2018 Mar 20;2018:1898421. doi: 10.1155/2018/1898421 (PMC5883977; doi:10.1155/2018/1898421)
Supplement: Supplementary Materials — Figure S1: DNA content of yeast cells differing in ploidy. Cellular DNA content of the yeast strains in the SP4, BY474X, and BMA64 genetic backgrounds. Propidium iodide-stained cells were analyzed via FACS as described in the Materials and Methods. Histograms were obtained for 10,000 cells per strain. Figure S2: ATP content and glucose uptake in yeast cells differing in ploidy. (A) ATP content was determined using BactTiter-Glo Microbial Cell Viability Assay. Luminescence was recorded using the microplate reader. The luminescent signal corresponded to the ATP content, which was directly proportional to the number of cells. (B) Glucose concentration was determined in YPD medium supplemented with glucose (20 mg/mL) using the Somogyi-Nelson method. The YPD medium was collected after 4 hours of cell incubation and used for analysis. Absorbance was recorded using the microplate reader at λ = 520 nm. The results are presented as the mean values from three independent experiments. The bars indicate SD. The stars indicate values that are significantly different from values obtained for haploid strain (1n) within the same genetic background using one-way ANOVA and Dunnett's post hoc test for SP4 and BY474X strains or t-test for BMA64 strain; ∗P < 0.05; ∗∗P < 0.01; and ∗∗∗P < 0.001, respectively. Figure S3: comparison of relative RNA content in yeast differing in ploidy. Relative RNA content of yeast cells was assessed with acridine orange. Fluorescence was examined under the fluorescence microscope at λex = 488 nm and λem = 650 nm. For determination of the relative value of RNA, images were separated into individual color channels; fluorescence intensity was measured only for the red channel using the cellSens Dimension software: (A) overlay; (B) red channel; (C) green channel. The results are presented as (D) the mean values of the fluorescence intensity for at least 100 cells from three independent experiments or as (E) calculations of the fluorescence intensity per cell s [file 1898421.f1.pdf]

Supplementary Materials

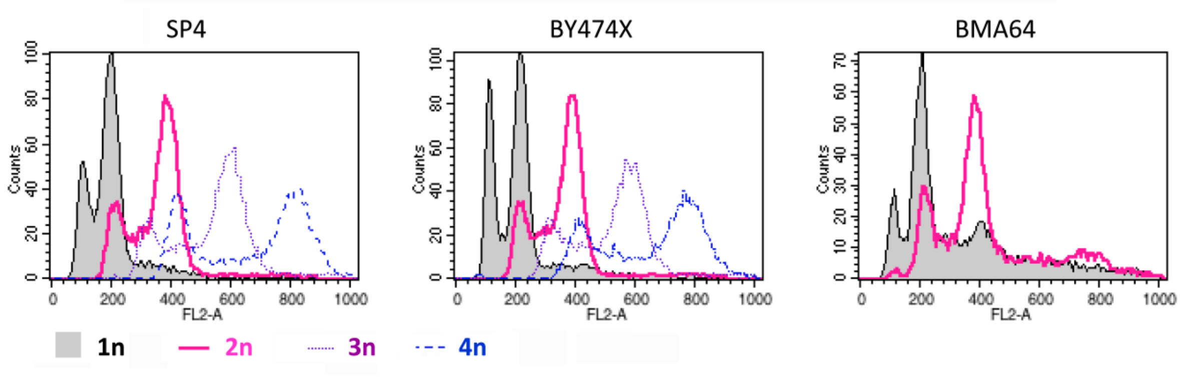

Figure S1. DNA content of yeast cells differing in ploidy. Cellular DNA content of the yeast strains in the SP4, BY474X and BMA64 genetic backgrounds. Propidium iodide-stained cells were analyzed via FACS as described in the Materials and Methods. Histograms were obtained for 10000 cells per strain.

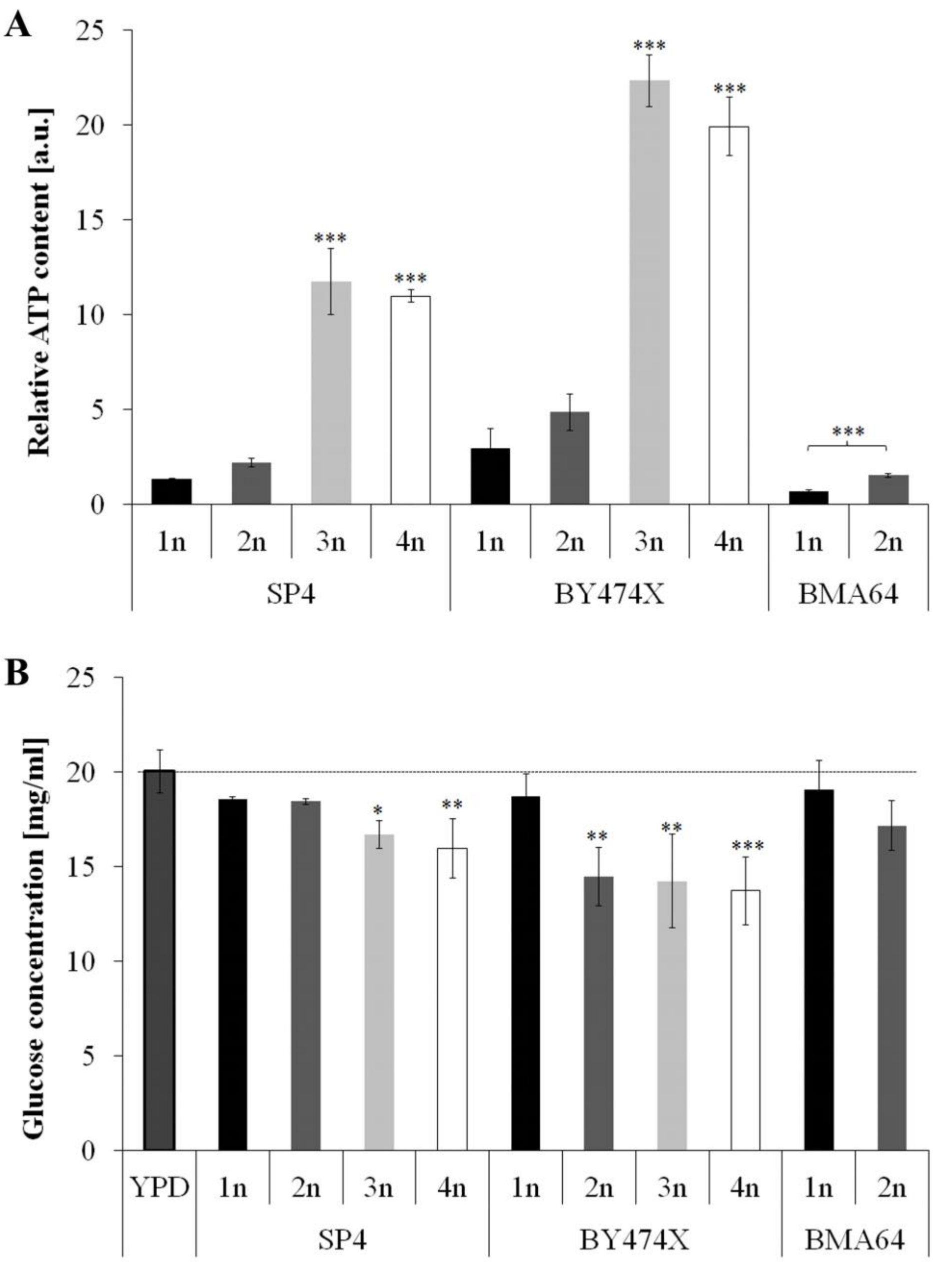

Figure S2. ATP content and glucose uptake in yeast cells differing in ploidy. (**A**) ATP content was determined using BactTiter-Glo™ Microbial Cell Viability Assay. Luminescence was recorded using the microplate reader. The luminescent signal corresponded to the ATP content, which was directly proportional to the number of cells. (**B**) Glucose concentration was determined in YPD medium supplemented with glucose (20 mg/mL) using the Somogyi-Nelson method. The YPD medium was collected after 4 hours of cell incubation and used for analysis. Absorbance was recorded using the microplate reader at $\lambda = 520$ nm. The results are presented as the mean values from three independent experiments. The bars indicate SD. The stars indicate values that are significantly different from values obtained for haploid strain (1n) within the same genetic background using one-way ANOVA and Dunnett's *post hoc* test for SP4 and BY474X strains or *t*-test for BMA64 strain; *$P < 0.05$; **$P < 0.01$; ***$P < 0.001$, respectively.

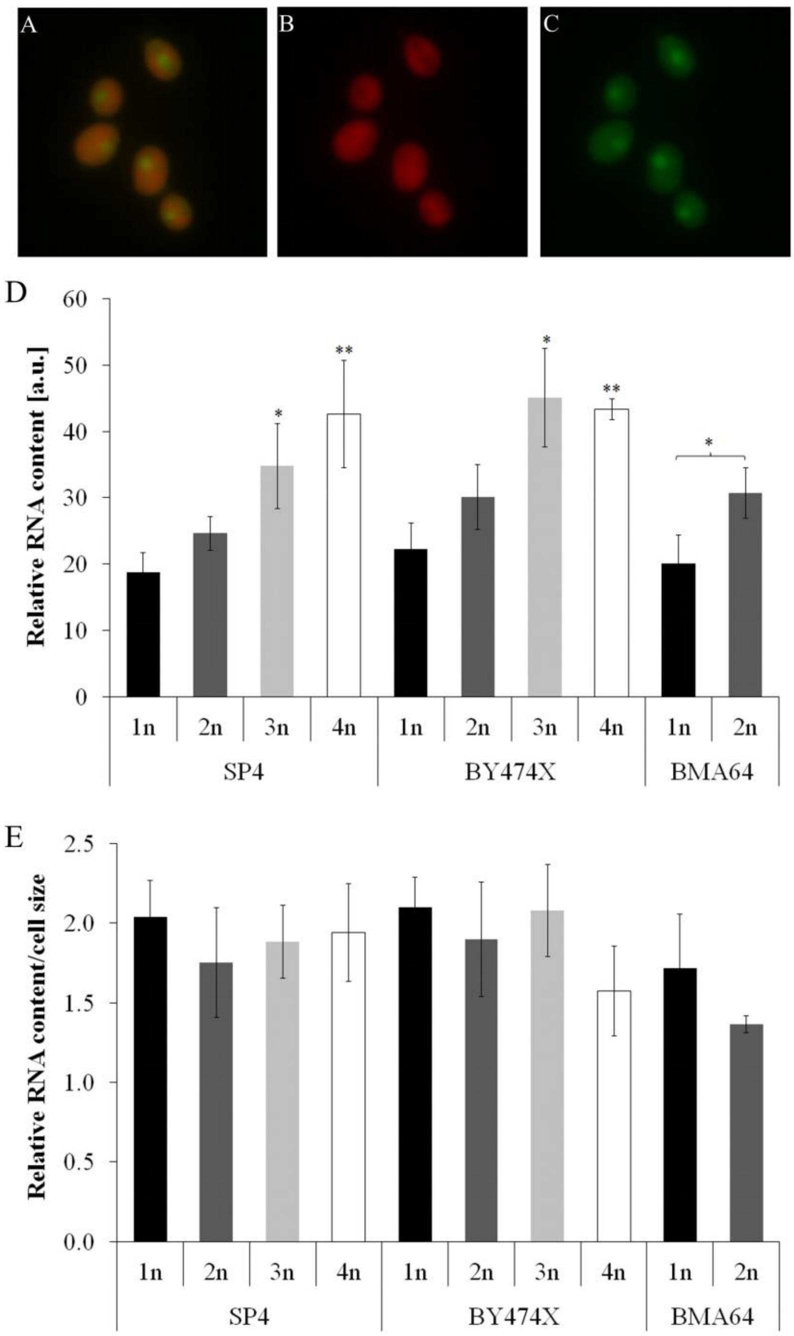

Figure S3. Comparison of relative RNA content in yeast differing in ploidy. Relative RNA content of yeast cells was assessed with acridine orange. Fluorescence was examined under the fluorescence microscope at λ_{ex} = 488 nm and λ_{em} = 650 nm. For determination of the relative value of RNA, images were separated into individual color channels; fluorescence intensity was measured only for the red channel using the cellSens Dimension software: (**A**) overlay; (**B**) red channel; (**C**) green channel. The results are presented as the mean values of the fluorescence intensity for at least 100 cells from three independent experiments (**D**) or as calculations of the fluorescence intensity per cell size (**E**). The bars indicate SD. The stars indicate values that are significantly different from values obtained for haploid strain (1n) within the same genetic background using one-way ANOVA and Dunnett's *post hoc* test for SP4 and BY474X strains or *t*-test for BMA64 strain; *P < 0.05; **P < 0.01; ***P < 0.001, respectively.

Yeast Strain		The part of the reproductive phase [%]
SP4	**1n**	26.8
	**2n**	26.8
	**3n**	49.3
	**4n**	50.0
BY474X	**1n**	20.0
	**2n**	25.5
	**3n**	34.9
	**4n**	23.1
BMA64	**1n**	30.0
	**2n**	62.0

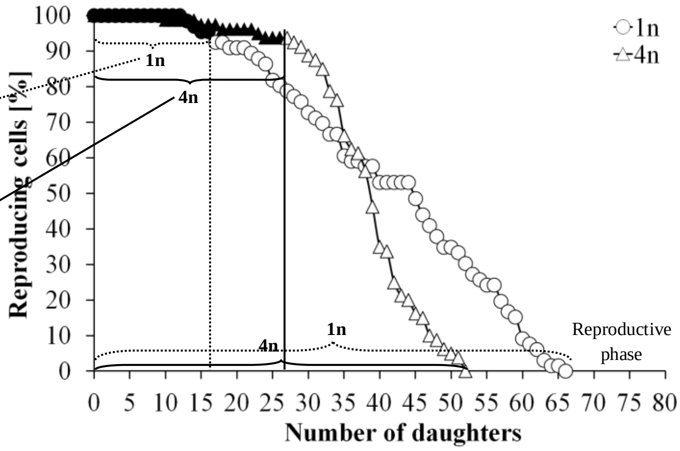

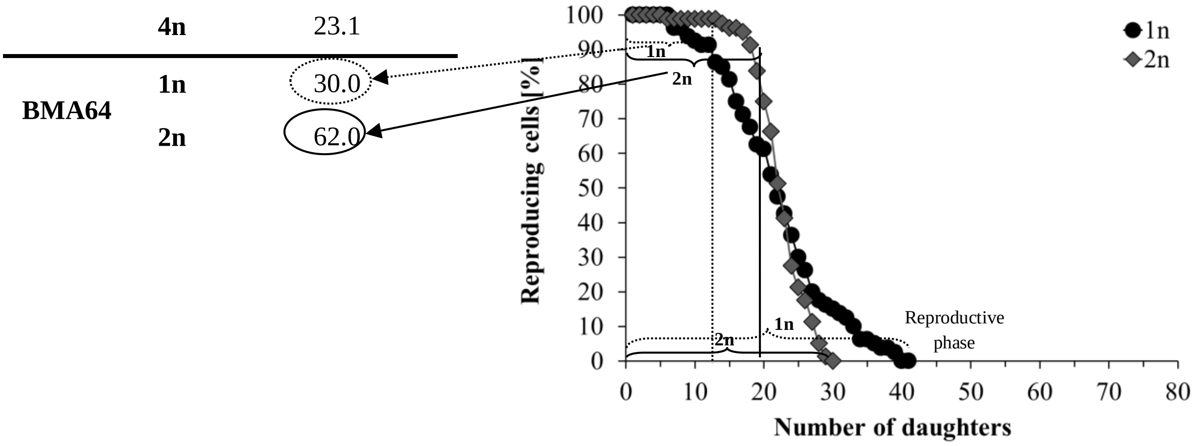

Figure S4. The impact of the number of genome copies on the reproductive possibility of the yeast cells. Calculations illustrating the part of the whole reproductive phase of life when almost all cells (90-100 percent) maintain the ability to reproduce. This value is strictly dependent on the number of the genome copies.

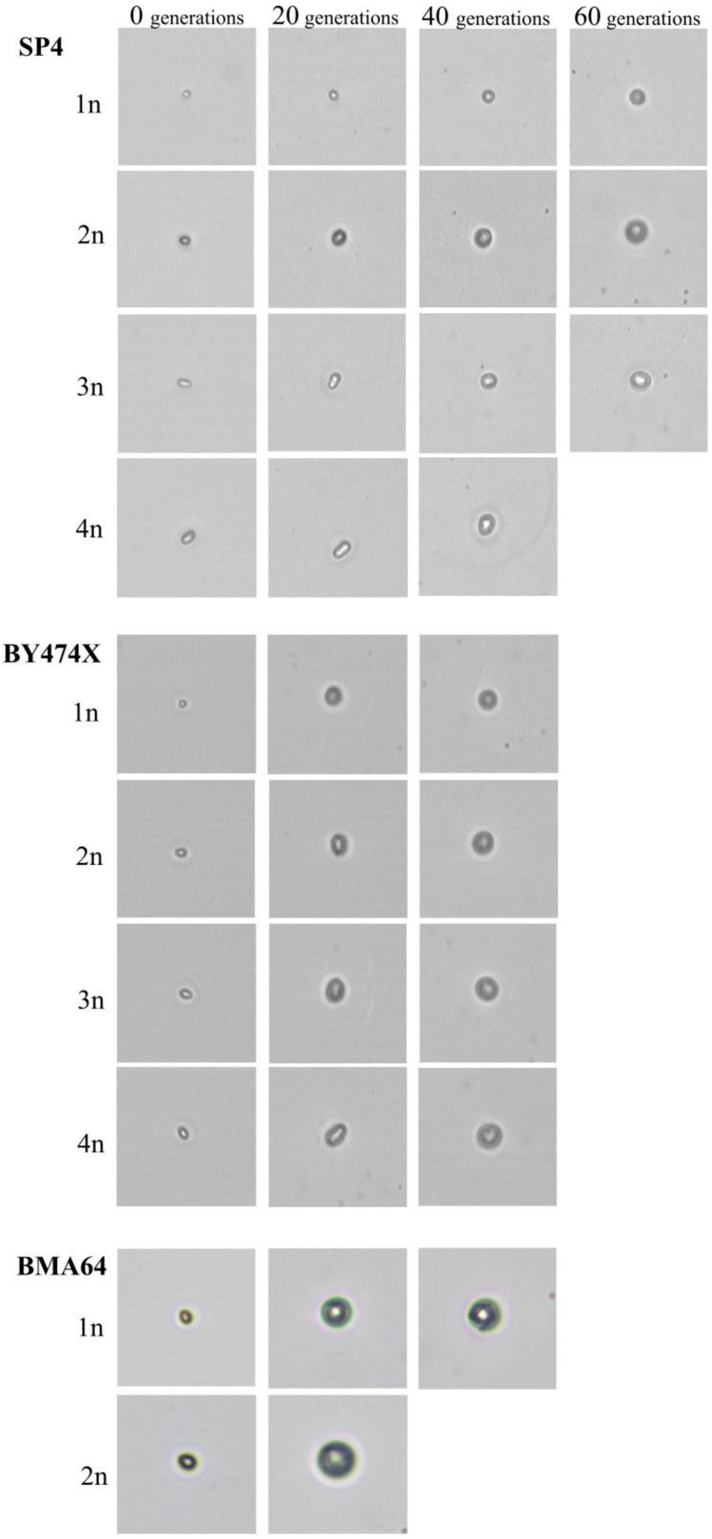

Figure S5. Changes in yeast cell shape during the reproductive phase of growth. Changes in the shape of the yeast cell during the reproductive phase of life were assessed by analysis of microscopic images recorded every fifth cell cycle during the reproductive potential determination procedure. The images are representative of all cells analyzed in two independent experiments. The analyzed yeast strains differ in ploidy (from 1n to 4n) and represent three genetic backgrounds: SP4, BY474X, and BMA64.
